# Current Trends in Approaches to Prevent and Control Antimicrobial Resistance in Aquatic Veterinary Medicine

**DOI:** 10.3390/pathogens14070681

**Published:** 2025-07-10

**Authors:** Dongqing Zhao, Konrad Wojnarowski, Paulina Cholewińska, Dušan Palić

**Affiliations:** Chair for Fish Diseases and Fisheries Biology, Faculty of Veterinary Medicine, Ludwig-Maximilians-University Munich, 80539 Munich, Germany; d.zhao@campus.lmu.de (D.Z.); k.wojnarowski@lmu.de (K.W.); p.cholewinska@lmu.de (P.C.)

**Keywords:** aquaculture, AMU, AMGs, bacteria, prevention

## Abstract

The growth of aquaculture production in recent years has revealed multiple challenges, including the rise of antimicrobial resistance (AMR) in aquatic animal production, which is currently attracting significant attention from multiple one-health stakeholders. While antibiotics have played a major role in the treatment of bacterial infections for almost a century, a major consequence of their use is the increase in AMR, including the emergence of AMR in aquaculture. The AMR phenomenon creates a situation where antibiotic use in one system (e.g., aquaculture) may impact another system (e.g., terrestrial–human). Non-prudent use of antibiotics in aquaculture and animal farming increases the risk of AMR emergence, since bacteria harboring antibiotic resistance genes can cross between compartments such as wastewater or other effluents to aquatic environments, including intensive aquaculture. Transferable antimicrobial resistance gene (AMG) elements (plasmids, transposons, integrons, etc.) have already been detected in varying degrees from pathogenic bacteria that are often causing infections in farmed fish (Aeromonas, Vibrio, Streptococcus, Pseudomonas, Edwardsiella, etc.). This review of current veterinary approaches for the prevention and control of AMR emergence in aquaculture focuses on the feasibility of alternatives to antimicrobials and supplemental treatment applications during on-farm bacterial disease control and prevention. The use of vaccines, bacteriophages, biosurfactants, probiotics, bacteriocins, and antimicrobial peptides is discussed.

## 1. Introduction

Aquaculture is a key industry in the world’s food supply, supporting livelihoods and optimal nutrition for billions of people. Seafood is rich in high-quality protein and healthy fats, has a variety of essential trace elements, and is a significant source of food worldwide. Aquaculture-produced seafood contributes over half of the total aquatic animal production, and as the scale of aquaculture continues to expand to meet the demand for aquatic animal food due to population growth, it is expected to reach 54% of global seafood production by 2032 [1]. Asia dominates the aquaculture sector, led by China (52.88 million tonnes), followed by India, Indonesia, Vietnam, Bangladesh, the Philippines, and South Korea. Outside of Asia, Norway, Egypt, and Chile complete the first ten aquaculture production countries on the list, and together these 10 countries account for 89.8% of total aquaculture production [1]. Species-wise, six finfish species (carp, tilapia, catfish, salmonids, bass, and snapper) contribute over 39.6 million tonnes (MMT) to aquaculture output [2].

Even though aquaculture significantly contributes to the global food supply, its potential is impacted by disease problems, often more than in other food-producing sectors [3]. High-density aquaculture farming is increasingly used as the preferred method of production, and while management advantages and economic benefits are obvious, such intensive culture methods inherently suffer from higher risks of the rapid spread of pathogens and the emergence of diseases. Disease outbreaks can increase economic losses as a consequence, and risk mitigation measures and treatments applied by veterinarians or other aquatic health professionals can assist in reducing the disease burden in production facilities [4]. Among the multitude of various fish pathogens (viral, bacterial, parasitic, fungal), bacteria such as Aeromonas, Vibrio, Streptococcus, Pseudomonas, and Edwardsiella [5,6,7] genera are of high prevalence and can cause significant economic losses.

A growing concern within this context is the emergence of the animal–human–environment interface, emphasized under the “One Health” framework. Aquaculture environments are increasingly situated near or in contact with human and/or animal fecal matter, creating ideal conditions for the emergence and spread of antimicrobial-resistant bacteria. Notably, resistant strains commonly associated with terrestrial animals, humans, and environmental sources are now being detected in fish populations as well. The application of antibiotics is common practice for the treatment of bacterial infections in aquaculture [8], and β-lactamases, tetracyclines, sulfonamides, macrolides, and fluoroquinolones are used most frequently [9]. The global aquaculture industry uses hundreds of tons of antibiotics each year and is expected to reach around 13,600 tons per year by 2030 [10]. The rapid rise of AMR has been hailed by the WHO, FAO and WOAH as one of the most important challenges for global one health, and specifically the human population in the XXI century [11]. What is even more troubling is that AMR can affect entire ecosystems through horizontal gene transfers between bacteria in different fish species or with environmental bacteria. Since aquaculture systems are at the interface of surrounding natural and man-made environments, AMG transfer risk is increased compared to terrestrial animal production [12]. Furthermore, bacterial strains isolated from urban wastewater environments can also show increased resistance to mercury, cadmium, chromium, and other common heavy metals [13,14]. With this phenomenon, the natural antibacterial properties of heavy metals can exercise selective pressure, which further promotes the development of resistant bacteria within already higher-risk aquatic environments. The aquaculture compartment therefore warrants increased attention in different initiatives aimed at mitigating the problem of antimicrobial resistance [15].

This article reviews the antibiotic resistance status in aquaculture from the perspective of aquatic veterinarians, analyzes the spread and harmful consequences of antibiotic resistance from the perspective of One Health and discusses alternatives to on-farm antibiotic use.

## 2. Spread of Antimicrobial Resistance in Aquatic Environments

The aquatic environment is a significant repository of AMGs [16,17]. Antibiotics from urban wastewater and sewage from residential and hospital buildings flow into the soil, which is the main pathway for antibiotics to enter underground aquatic compartments [18]. These antibiotics can exist in urban groundwater systems for a long time, thereby selecting and proliferating antibiotic-resistant bacteria (ARB) and AMGs in groundwater and increasing the possibility of horizontal gene transfer between pathogens [19].

Akaniro et al. detected that 86% of Aeromonas, Vibrio, Pseudomonas, and Salmonella isolates in groundwater samples from Nigeria were multidrug-resistant, showing high resistance to cefuroxime, amoxicillin, and ampicillin [20]. Aquaculture’s dependence on surface runoff and groundwater use allows these resistant bacteria, already selected by the environment, to directly enter the aquaculture environment [21]. *Bla*_TEM_ (β-lactam resistance), *sul* (sulfonamide resistance), and *tet* (tetracycline resistance) genes have been detected in coastal and estuarine waters that are strongly affected by human activities in different countries [22,23]. It is worth noting that *vanA* genes of vancomycin-resistant Enterococcus (VRE) were detected in urban sewage, livestock manure, and freshwater biofilms [24,25]. The presence of this gene, which was previously very rarely detected in the water environment, poses an even higher risk for human public health, as vancomycin remains the last line of defense against antimicrobial-resistant bacteria in clinical treatment [26].

Aquaculture is often combined with livestock farming and agriculture in integrated fish farms (especially in Asia), opening an additional pathway for AMGs to move from land to soil and, ultimately, to surface waters [27]. Lei et al. found that more than half of the AMGs and bacteria in livestock farms were flowing to groundwater along with livestock manure and soil, and as many as 45 kinds of AMGs, which were mainly resistant to aminoglycosides, sulfonamides, and tetracycline antibiotics, were found to flow into underground rivers and spread rapidly through water systems [28]. Some studies have found that the content of AMGs in waste discharged from livestock farms is significantly higher than that discharged from human activities [29]. This multi-drug resistance gene environment may have contributed to the emergence of the quinolone resistance gene *qnrA* [30], which is encoded by plasmids and stored by the aquatic bacteria Shewanella and Aeromonas and is widespread in both marine and freshwater environments [31,32]. In areas with high livestock density, resistance genes in groundwater can persist for many years, posing a long-term risk to regional groundwater and drinking water sources and allowing AMGs to circulate in the ecosystems formed by animals, humans, and the environment for a long time [33,34].

Intensive and semi-intensive production practices are common in fish production, which provides the possibility of AMGs spreading in host environments (such as fish gut microbiota). Due to interactions between complex virulence factors, secretion systems, and AMGs, pathogenic microorganisms and opportunistic pathogenic microorganisms carried by fish groups form a close relationship, which jointly promotes the risk of infection in fish populations [35]. In addition, in areas with long-term freshwater aquaculture, bacteria that carry resistance genes may pass through fish facilities (ponds, raceways, cages etc.) into open waters (lakes, rivers) and eventually into groundwater. Tong et al. reported the presence of AMGs in fishpond water, surface water, and groundwater of Honghu Lake, China [36]. This widespread presence may be partly explained by the findings of the WHO, which reported that antibiotics commonly used in agriculture and aquaculture are structurally related to those used in human medicine, promoting both co-resistance and cross-resistance [37]. Such processes further contribute to the growth and diversification of the resistance gene pool [38]. Supporting this, Wanyan identified the prevalence of specific resistance genes such as *aadA-02* and *floR* in Chinese aquaculture sediments [39]. Moreover, antibiotics like tetracycline tend to adsorb to sediments, allowing them to persist in these environments for extended periods [40]. As a result, sediments may serve as long-term reservoirs of antibiotic resistance genes, posing ongoing risks to aquatic ecosystems and organisms. Sargenti et al. found abundant residues of quinolones (ciprofloxacin, flumequine, and olinic acid) and tetracycline (oxytetracycline) in sediments sampled near Italian fish farms [41].

**Figure 1:**
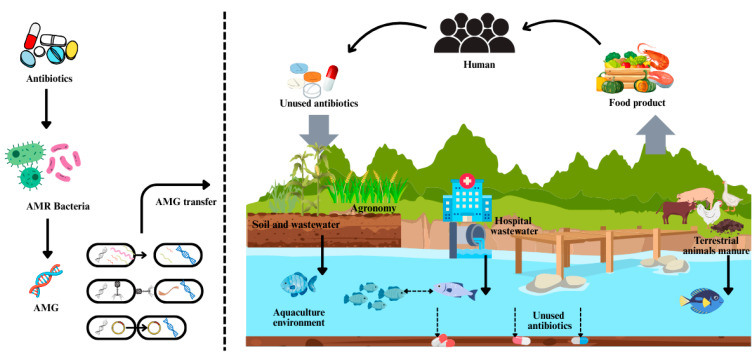
AMR bacteria and AMG connectivity between aquaculture, terrestrial, human and other compartments (based on [12,13,14,15,16,17,18,19,20,21,22,23,24,25,26,27,28,29,30,31,32,33,34,35,36,37,38,39,40,41]).

AMR bacteria carrying AMGs can easily enter the environment through soil, hospital wastewater and animal manure and eventually pollute the water and aquaculture environment, indirectly affecting the existence of normal bacterial flora in these ecological niches. As it further spreads in these systems, fish consumers may ingest antibiotics unknowingly, leading to a range of antibiotic allergy and toxicity issues (Figure 1) [42].

## 3. Prevalence of AMR and AMGs in Fish

### 3.1. Aeromonas

*A. salmonicida*, *A. hydrophila*, and *A. veronii* are the most common Aeromonas bacterial species that infect and are clinically isolated from fish outbreaks worldwide [43], infecting a variety of farmed fish species and causing huge production losses. Aeromonas infection presents with a range of clinical symptoms, including skin ulcers, edema and bleeding in farmed fish in freshwater and marine environments [44,45]. In addition, bacteria from the genus Aeromonas can produce a variety of virulence factors. The aerolysin gene (*aer*) is the most common virulence gene, leading to disruption of membrane permeability and cell death by inducing pore or channel formation [46]. The hemolysin gene (*hlyA*) and enterotoxin gene (*act*) were also detected in *Aeromonas* isolated from diseased fish [47,48]. Virulence factors are usually related to the synthesis of hemolysins, lipases, proteases, adhesins, as well as biofilm formation, and infect fish by regulating the expression of pathogenic factors through quorum sensing [49].

*A. salmonicida* can cause fish furunculosis and salmonids are considered primary hosts [50]. However, individual fish can exhibit varying degrees of resistance to *A. salmonicida*, and some fish carrying resistance genes can survive disease outbreaks and become reservoirs of infection [51]. Wojnarowski et al. identified AMGs (*mcr-3*, *fox-2*, *cphA5*, *and OXA-427*) in *A. salmonicida subspecies salmonicida* clinical isolates collected during a clinical outbreak on brook trout and char farms in Bavaria, Germany, which may have been transmitted via horizontal transfer [52]. Class 1 integrons carrying multiple gene cassettes have been identified in *Aeromonas* plasmids, which can carry resistance genes such as *dfr*, *aadA* and *cat* and are associated with MDR formation [53]. Ponce et al. found that *A. salmonicida* isolated from salmon gut in Chilean farms carries the *dfrA*14 gene cassette and confers resistance to both sulfonamides and trimethoprim [54].

In Egypt, 53.4% of *A. hydrophila* isolates from diseased Nile tilapia fish had some AMGs, with 69.0% of the isolates exhibiting multidrug resistance (chloramphenicol, amikacin, gentamicin) [55]. Additionally, 46% of *A. jandaei*, 31% of *A. veronii*, and 11% of *A. hydrophila* were reported in clinically asymptomatic freshwater fish in Malaysia, with a number of MDRs (*sul1*, *strA-strB*, *aadA*, *bla*_TEM_, *bla*_SHV_, *tetA*-*tetE*, and *tetM*) [56]. *A. veronii* can attack a variety of fish species, including African catfish (*Clarias gariepinus*) [57], Mandarin fish (*Siniperca chuatsi*) [58] and European seabass (*Dicentrarchus labrax*) [59].

### 3.2. Vibrio

*Vibrio* spp., with *V. harveyi*, *V. anguillarum* and *V. vulnificus* mostly identified as causatives, can present with necrotic syndrome, acute gastroenteritis and septicemia [60,61,62]. *V. harveyi* is the main pathogen of luminous vibriosis in shrimp [63], and recently *V. harveyi* was isolated from ulcerated skin lesions in dead Spotted Sea Bass (*Lateolabrax maculatus*), where resistance to cephalosporin (vancomycin, cefoperazone, cefradine) and aminoglycoside (piperacillin) was first detected [64]. Li et al. isolated a strain of *V. anguillarum* from *Sebastes schlegelii* suffering from red body disease, which was resistant to eight drugs, including β-Lactams (*carB-19*, *mecB*), Aminoglycosides (*novA*) and Quinolones (*QnrS2*) [65].

In most cases, the antibiotic resistance of *Vibrio* is not inherent to plasmids but is due to the fact that the genes encoding resistance are located on the chromosome [64,65]. However, a plasmid-encoded carbapenemase was found in *V. parahaemolyticus* isolated from yellowstripe scad (*Selaroides leptolepis*), Indian mackerel (*Rastrelliger kanagurta*), black pomfret (*Parastromateus niger*), catfish (*Clarias batrachus*), and red tilapia (*Oreochromis* spp.) [66], and plasmid-mediated quinolone resistance (PMQR) genes *qnr* were detected in *V. anguillarum* isolated from Korean mullet (*Mugill cephallus*) aquaculture [67]. In fact, reduced susceptibility to quinolones has been mainly attributed to point mutations in QRDR, and nonsynonymous substitutions in *gyrA*, *gyrB* and *parC* genes in QRDR have been reported in *V. vulnificus* isolated from Asian sea bass (*Lates calcarifer*) farms [16], which poses a serious threat to the spread of resistance genes in aquaculture. *V. cholerae* is also a major concern for the public, with a high mortality rate in human infection but a low prevalence in aquaculture and its products, and most of them belong to non-O1/O139 and non-O141 serogroups [68]. However, *V. cholerae* recently isolated from freshwater fish in China was multidrug resistant to streptomycin, ampicillin and rifampicin (57.6%) [69].

### 3.3. Streptococcus

Streptococcus species most frequently isolated from farmed fish include *S. agalactiae*, *S. iniae* and *S. parauberis*. The most harmful of these is *S. iniae*, which, in addition to the emergence of antibiotic-resistant strains, also has a large number of virulence genes that enable it to enter, multiply and evade the immune system of fish and other aquatic organisms [70]. It has been documented that *S. iniae* can infect 27 species of fish, including Gilthead Sea bream (*Sparus aurata*), Rainbow trout (*Oncorhynchus mykiss*) and Tilapia (*Oreochromis niloticus*) [71]. *S. iniae* isolated from diseased Tilapia in Egypt is highly resistant to erythromycin (*ermB*) and tetracycline (*tetM*, *tetO*) [72]. *S. iniae* can also increase resistance to oxytetracycline and florfenicol by forming biofilms [73]. *S. agalactiae* is mainly associated with high mortality in tilapia and carries the *tetM* and *ermB* genes. Interestingly, serotypes Ia and III of *S. agalactiae* show opposite resistance to erythromycin, which may be due to differences in bacterial capsules between different genomes, leading to different levels of horizontal transfer of erythromycin resistance genes [74]. *S. parauberis* was first detected in cultured turbot (*Scopthalmus maximus* (L.)) that broke out in Spain [75]. Subsequently, *S. parauberis* isolated from olive flounder and starry flounder showed that the distribution of resistance genes was significantly correlated with its serotypes. *ermB*, *tetS* and *ANT (6)-Ia* were only found in the serotype Ia genome, while *tetM* and *mef (J)-msr (I)* were only present in serotype II [76]. *S. uberis* was recently isolated from moribund mandarin fish (*Siniperca chuatsi*), which can cause serious pathological changes in the brain tissue of Mandarin fish and grass carp [77]. *S. uberis* was originally isolated from cows with mastitis [78] and has a fatality rate of up to 60% in Mandarin fish. This kind of disease caused by contact with fecal-contaminated water should not be underestimated in aquaculture.

### 3.4. Pseudomonas

The *Pseudomonas* genus is composed of more than 200 ubiquitous species, most commonly isolated from fish undergoing putrefaction processes [79]. Among them, *P. putida* is pathogenic to important economic fish species such as Nile tilapia (*Oreochromis niloticus*) [80], Common Carp (*Cyprinus Carpio*) [81], sea bass (*Dicentrarchus labrax*) [82] and Rainbow Trout (*Oncorhynchus mykiss*) [83]. *P. aeruginosa* is widely present in aquatic environments as an opportunistic pathogen, which can transform from a commensal form to a highly virulent form and is believed to cause fish septicaemia [84]. The most common antibiotic resistance genes in *P. aeruginosa* isolated from fish are *bla*_TEM_, *tetA*, and *bla*_CTX-M_, and multi-resistance is common [85]. *oprL* and *toxA* genes are the most dominant virulence genes, associated with outer membrane protein synthesis and inhibition of protein synthesis in host cells, respectively [84]. *P. fluorescens* isolated from Nile tilapia in Egypt showed resistance to piperacillin, ceftazidime and cefepime [86], which is closely related to the natural resistance of *Pseudomonas* species to beta-lactam antibiotics. *P. baetica* was originally isolated from Spanish wedge sole (*Dicologlossa cuneata*) [87]. Recently, a type 1 integron was discovered in the *P. baetica* strain isolated from salmon gut, carrying three AMGs (aminoglycosides (*aac[6′]-31*), Beta-lactams (*bla*_OXA-2_), and quaternary ammonium compounds (*qacH*)) [54]. This integron is associated with a plasmid and can promote the spread of AMGs, which poses a potential risk to salmon farms because some disinfectants commonly used in the food industry are quaternary ammonium compounds [88].

### 3.5. Flavobacterium

In addition to the above-mentioned bacterial pathogens commonly reported in fish culture, *Flavobacterium* species are also one of the major fish pathogens. It has been reported that *F. columnare*, *F. psychrophilum*, and *F. branchiophilum* are three major species of *Flavobacterium* that can cause fish infections, with freshwater fish being more susceptible [89]. Kumru et al. identified antimicrobial resistance genes to 11 classes of antibiotics (aminoglycosides (*AGly*), beta-lactamases (*bla*), cationic peptides (*col*), fluoroquinolones (*flq*), glycopeptides (*gly*), macrolide-lincosamide-streptogramin (*mls*), oxazolidinones (*oxzln*), phenicols (*phe*), sulfonamides (*sul*), tetracyclines (*tet*), and trimethoprim (*tmt*)) in aquatic *Flavobacterium* species, encoding at least one P-type IV secretion system (T4SS) protein [90], which may facilitate the spread of these AMGs [91]. Declercq et al. also found proteins encoded by three virulence genes *AraC*, *NodT*, and *LuxR* in the genome of trout *F. columnare* isolates [92]. The *NodT* family proteins are a group of resistant nodule cell division (RND) type efflux systems, which play an important role in the intrinsic and acquired multidrug resistance of *F. columnare* [93].

### 3.6. Acinetobacter

*Acinetobacter* is persistent and widely distributed in the environment and is also facultative pathogens. Recently, multiple species of *Acinetobacter* have been identified as fish pathogens causing disease outbreaks in farmed fish, including *A. johnsonii* and *A. lwoffii* infecting Rainbow Trout (*Oncorhynchus mykiss*) [94,95], *A. pitti* infecting bighead catfish (*Clarias. macrocephalus*) [96], and *A. baumannii* infecting *Pagellus acarne* [97]. Infected fish show sepsis symptoms, varying degrees of ascites, and systemic ulcers [98]. In particular, *A. baumannii* can be resistant to almost all antimicrobial drugs and is one of the common nosocomial pathogens [99]. Hasiri et al. isolated *A. baumannii* from fish and shrimp, with high resistance rates to tetracycline, ampicillin, gentamicin and erythromycin, and carried *bla*_CITM_, *bla*_SHV_, *tetA*, *qnrA*, *bla*_VIM_ and *aac(3)-IV* genes [100]. In addition, a new MDR strain of *A. pittii* was found in Indian rohita (*Labeo rohita)*, which was resistant to ampicillin, cefoxitin, cephalexin, nitrofurantoin, methoxycillin, penicillin-G, bacitracin and methoxazole [101]. In contrast to the extensive multidrug resistance of *A. baumannii*, *A. lwoffii i*s still sensitive to almost all antibiotics, which may be because T4SSs encoding plasmids are more common in *A. baumannii* and can promote antibiotic resistance and biofilm synthesis via conjugation and the secretion of proteins [102,103].

### 3.7. Edwardsiella

*Edwardsiella* spp. are widely distributed throughout the world, with three important fish pathogens (*E. ictalurid* [104], *E. piscicida* [105] and *E. anguillarum* [106]), reported in many countries in Europe, Asia and the United States, causing infections in more than 20 species including channel catfish (*Ictalurus punctatus*), blue catfish (*I. furcatus*), red sea bream (*Pagrus major*), and European eel (*Anguilla anguilla*). Gastrointestinal diseases, blood-borne infections, and wound infections are the most common clinical manifestations [107]. Islam et al. discovered plasmid pEIMS-18199 from *E. ictaluri* of channel catfish, containing *floR*, *tetD*, and *sul2* genes, which conferred resistance to florfenicol, tetracycline, and sulfonamides [108].

In fact, pEIMS-171561 belongs to the incompatibility group A/C (IncA/C), and the IncA/C group is one of the earliest plasmids associated with antimicrobial resistance that is often associated with the spread of multiple clinically relevant resistance genes, including *bla*_CMY_, *bla*_NDM_, and *sul2* [109]. The IncA/C group plasmids are self-transferable and have been identified in many bacterial species such as *A. hydrophila*, *A. salmonicida*, *V. cholera* and *E. coli* [110].

*E. piscicida* may also have the ability to transmit multidrug resistance genes to other bacteria. For example, the plasmid pEPMS-18199, which contains six genes known to confer resistance to florfenicol (*floR*), tetracycline (*tetA* and *tetR*), sulfonamide (*sul2*), and aminoglycoside (*aph(6)-Id (strB)* and *aph(3)-Ib (strA)*), was found in an *E. piscicida* strain recovered from hybrid catfish and was highly stable in the host cell and could be transferred to *E. coli* and *E. ictaluri* by conjugation [111].

*E. anguillarum*, as an atypical non-motile fish pathogen, was distinguished from *E. tarda* classification in 2016 [106]. Currently, most studies are focused on its pathological characteristics and virulence analysis, and only a few studies have been conducted on its resistance genes [112,113]. However, *E. anguillarum* recently isolated from milkfish and Japanese eels was found to contain a unique genomic island (GI), which is part of the genome that may undergo horizontal gene transfer after acquiring multiple virulence genes, resistance genes or the ability to adapt to a specific environment [114]. *E. tarda* was originally isolated from humans but has a broad host range in fish species [115]. In addition, *E. tarda* can carry naturally occurring R plasmids that confer resistance to sulfonamide and tetracycline, or sulfonamide, streptomycin, chloramphenicol, tetracycline, and kanamycin [116]. This conclusion was confirmed by the detection of *bla*_TEM_, *sul1*, *tetA*, *bla*_CTX-M_, *aad1*, *qnrS* and *qnrA* resistance genes in *E. tarda* isolated in Egypt [117].

### 3.8. Other Clinical Strains

Methicillin-resistant *Staphylococcus aureus* (MRSA), which is commonly seen in human clinical trials, was found in marine fish—dusky kob (*Argyrosomus japonicus*)—and 82% of strains were recorded as MDR strains, with *femA*, *blaZ*, *tetA*, *tetM*, and *ermB* genes being widespread [118]. Jinnai et al. detected extended-spectrum β-lactamase (ESBL)-producing *E. coli* and *K. pneumoniae* in the intestinal contents of edible river fish from Vietnam, containing transferable plasmids encoding *bla*_CTX-M-15_, *bla*_CTX-M-27_, and *bla*_CTX-M-55_ [119]. The emergence of these “superbugs” in fish is an emerging cause for concern. Table 1 summarizes the key findings on AMR and AMGs in fish-associated bacteria.

## 4. Alternative Strategies

Because of the high-density farming environment in intensive aquaculture systems, the possibility of pathogens spreading between individual fish is significantly increased. Although various biosecurity and management measures, including isolation/quarantine, equipment disinfection, use of high-quality feed, water treatments, and proper disposal of dead fish, have been shown to be effective in reducing disease transmission, these measures cannot always eliminate the risk of pathogens and disease outbreaks. Frequent metaphylactic, and more importantly, non-prudent use of antibiotics in aquaculture can increase the risk of antibiotic resistance emergence, rendering the use of selected or multiple antibiotic disease treatments ineffective, followed by increased antibiotic residues in fish products [100]. Some environmentally friendly and economical alternatives and supplemental treatment options that would be applicable in an effort to minimize the use of antibiotics in aquaculture are explored below.

### 4.1. Vaccines

Vaccination programs against bacterial pathogens are a time-tested preventive tool, and in cases where an alternative to antibiotics is required to control disease outbreaks, autogenous vaccines can be applied as part of the disease control strategy. In the actual application of aquaculture, many important factors need to be considered, including fish species, immune system status, administration route, nutrition and cost-effectiveness [123]. Among the preventative measures gaining increased use in aquaculture are autogenous vaccines, which are inactivated, non-commercial biological products derived from pathogens isolated during site-specific disease outbreaks [124]. These vaccines are intended for exclusive use within the same epidemiological unit from which the pathogenic strain was obtained. They are produced under veterinary supervision and do not undergo the same centralized authorization process as commercial vaccines, which allows for a faster turnaround time in the face of localized disease challenges. The primary advantage of autogenous vaccines lies in their high degree of antigenic match to the circulating strain, which ensures improved immunological relevance and effectiveness compared to broad-spectrum commercial vaccines. This specificity is especially important in aquaculture settings, where strain variation between geographic regions or even individual farms can limit the efficacy of standard vaccines. As such, autogenous vaccines can serve as a rapid, targeted response when commercial products are unavailable, insufficiently protective, or not yet developed [124]. By inducing immunity against locally prevalent pathogens, autogenous vaccines contribute significantly to reducing disease incidence and severity in farmed fish populations. Although they may not fully eradicate infections, they are instrumental in slowing down the spread of pathogens, lowering pathogen load in the environment, and minimizing the overall impact of outbreaks [125]. This mitigation effect can be particularly important in high-density aquaculture systems, where infectious agents can spread rapidly and lead to severe economic losses.

Despite their benefits, autogenous vaccines also face limitations, including variability in production quality, lack of standardized protocols across regions, and limited regulatory oversight in some countries. Nevertheless, when used as part of a broader disease management strategy, they represent a valuable tool for enhancing aquatic animal health and reducing the need for antibiotic treatments, thereby contributing to efforts to control antimicrobial resistance in aquaculture systems [125]. Moreau et al. compared the effectiveness of autogenous vaccines against furunculosis caused by *Aeromonas salmonicida* subsp. *salmonicida* in large Rainbow trout (*Oncorhynchus mykiss*) using intraperitoneal injection, immersion, and oral administration via food, and found that only the regimen involving at least one intraperitoneal injection was truly effective [126]. Although oral and immersion vaccination have been repeatedly shown to be effective ways of administration [126], the difference in results may be due to the fact that immersion vaccination is more effective in smaller salmonids, while the acidity of the stomach may destroy most of the ingested antigens when administered orally. Ahmadivand et al. developed a soluble IHNV glycoprotein on self-assembled ferritin nanoparticles, which can exhibit high stability under low pH conditions in the gastrointestinal tract of trout [127].

Approved commercial fish inactivated vaccines can prevent infections caused by various pathogens such as *Lactococcus*, *Streptococcus*, *Aeromonas*, and others. However, inactivated microorganisms typically induce weaker or shorter-lasting immunity compared to other vaccine types, and the use of adjuvants may increase the risk of local or systemic adverse reactions, particularly after multiple booster vaccinations.

Live attenuated vaccines, which utilize bacteria or viruses with reduced or removed pathogenicity, are used in aquaculture to prevent diseases such as furunculosis, vibriosis, *Edwardsiella* infections, and others. These vaccines generally induce a stronger immune response than inactivated formulations, often mimicking the effect of natural infection and providing longer-lasting protection [128]. For example, a live attenuated vaccine developed by knocking out the virulence gene hisJ of *A. hydrophila* was shown to protect loach (*Misgurnus anguillicaudatus*) against infection and to increase levels of specific IgM antibodies and cytokines [129].

Despite these advantages, live attenuated vaccines carry inherent safety concerns that must be carefully considered. One of the primary risks is reversion to virulence, where the attenuated strain may regain pathogenic potential through spontaneous mutation, recombination with wild-type strains in the environment, or acquisition of mobile genetic elements. This is especially relevant in open or semi-open systems, where environmental bacteria may facilitate horizontal gene transfer. Additionally, immunocompromised fish may be more vulnerable to unintended pathogenic effects even from attenuated strains [130]. Although such events are rare, they underscore the importance of vaccine design and post-deployment monitoring. The long-term success of the AQUAVAC-ESC^®^ (Merck Animal Health, Rahway, NJ, USA) live attenuated vaccine (formerly USDA, ARS, RE-33), used for over a decade in U.S. catfish farming without reported reversion to virulence, demonstrates that attenuated vaccines can be safe and effective when properly developed and implemented [129]. Nevertheless, their use should be accompanied by strict biosecurity protocols, and environmental persistence of vaccine strains should be assessed during development to minimize ecological and health risks.

Subunit vaccines use only the key antigenic components of pathogens, and the main advantage is high safety. Because they cannot proliferate in the host, their ability to stimulate immune responses may be weaker than inactivated vaccines; therefore, repeated vaccinations are required to enhance the effect. One of the methods for producing subunit vaccines is to purify them directly from the target pathogen or use recombinant expression vectors to produce specific immunogenic proteins [130]. Recombinant vaccines developed using outer membrane proteins (OMPs) as candidate antigens have been immunologically evaluated in a variety of fish species [131]. In addition, DNA vaccination has been studied in a variety of fish. It is based on the administration of a plasmid that encodes the vaccine against the antigen, rather than the antigen itself. Hu et al. constructed the optimized MCP gene into the pVAX1 vector to construct the pV-FoptiMCP recombinant vector and developed a DNA vaccine against Largemouth bass virus (LMBV). The protection rate against largemouth bass (Micropterus salmoides) was as high as 89% four weeks after the initial immunization [131]. Sun et al. compared the immune effects of a subunit vaccine induced by purified recombinant TssJ (a functional gene of the T6SS secretion system) and a DNA vaccine encoding TssJ antigen against *V. harveyi* for golden pompano (*Trachinotus ovatus*), and found that although both vaccines induced upregulation of multiple immune-related genes such as IL10, C3, MHC Iα and IgM, the DNA vaccine caused a higher expression level of these genes [132]. This may be because DNA vaccines can stimulate both exogenous and endogenous pathways of antigen delivery, such as stimulating immune-presenting cells to phagocytose and take up apoptotic bodies, which in turn can induce stronger specific humoral and cellular immune responses in fish than other types of vaccines [133].

Currently, we can observe that more and more innovative technologies are being used in aquaculture; one such development would be nanoparticle vaccines, which are used as a novel vaccine design platform, offering the possibility of efficient targeted delivery, providing antigen stability and immunogenicity, with especially significant commercial potential for enveloped viruses [127]. Given the potential presence of multiple potential pathogens in the aquaculture environment, the use of multivalent or multi-combination vaccines is considered a highly cost-effective approach to effectively protect against infection by common pathogens such as *V. alginolyticus*, *V. harveyi* and *S. iniae* [134]. In addition, plant-based fish vaccines, which utilize plants to produce recombinant proteins, also have great potential in reducing the dose of pathogen boosters due to their low cost, edibility, and ability to be post-translationally modified [135].

Currently, the regulatory environment of vaccines in aquaculture faces many challenges, which are mainly due to the diversity of vaccine types, the complexity of application environments, and the differences in safety, effectiveness and controllability of different vaccines. Although autogenous vaccines can be customized according to pathogens isolated from specific farms and they have the advantages of high specificity and rapid response, the standardization of production process quality control and assurance has been generally missing across EU member states due to inherent issues such as pathogen variability, different field conditions, and vaccine application techniques [126]. Inactivated vaccines and live attenuated vaccines are more mature in commercialization. However, the immune efficacy of inactivated vaccines is weak, and the duration is short. Adjuvants or multiple booster injections are often required, which may cause local or systemic adverse reactions, especially under high-density farming conditions, which increases the difficulty of regulation. Although live attenuated vaccines can induce stronger immune responses, their potential pathogenicity risks and their safety still need to be continuously tracked and evaluated [136]. New vaccines such as subunit vaccines and DNA vaccines are favored due to their higher safety, but they generally have the problem of insufficient immunogenicity and require repeated vaccination or the use of advanced delivery systems (such as nanoparticles) to improve their effectiveness, which places higher demands on vaccine quality control and production consistency [137].

### 4.2. Bacteriophages

Bacteriophages are viruses that specifically infect and lyse bacteria, and they have re-emerged as targeted antimicrobial agents with increasing potential for application in aquaculture systems. Abundant in marine and freshwater ecosystems, phages demonstrate a high degree of host specificity and the ability to replicate only in the presence of their bacterial targets. This selectivity, combined with their minimal impact on commensal microbial communities, makes them attractive candidates for disease control in aquatic species [138,139].

In aquaculture, several studies have validated the effectiveness of phage therapy against key bacterial pathogens, including *V. anguillarum* [140], *V. harveyi* [141], *A. salmonicida* [142], and others [143]. These infections, common in farmed fish species, have shown significant reduction in both mortality and bacterial load following phage treatment [144]. Notably, targeted phage applications have proven particularly effective in preventing *Yersinia ruckeri* outbreaks in salmonids during stressful operational events such as grading, vaccination, and transport, where bacterial shedding and disease risk are elevated [145].

Laboratory experiments consistently support the superior performance of phage cocktails over monophage therapies. Blends of multiple phages typically result in faster bacterial inactivation and a reduced likelihood of resistance emergence [146]. In controlled in vivo trials, phage therapy effectively mitigated infections caused by multidrug-resistant (MDR) pathogens, further supporting its utility in the face of rising antimicrobial resistance in aquatic settings [147].

Nonetheless, resistance development remains a significant challenge to long-term phage efficacy. Bacterial populations may evolve various defense mechanisms, including modifications to phage receptors, biofilm enhancement, or secretion of proteases that degrade phages [148]. Recent research has explored strategies such as phage–antibiotic synergy (PAS), wherein co-administration of certain antibiotics (e.g., ceftazidime) with phages results in amplified bacterial suppression and delayed resistance. These effects are partly attributed to antibiotic-induced morphological changes in bacterial cells that facilitate phage replication [149,150].

Beyond the biological complexities, the commercial use of phages in aquaculture faces major production and regulatory hurdles. Industrial-scale phage production involves cultivating bacterial hosts, followed by complex purification steps to eliminate endotoxins, cellular debris, and non-viable viral particles. Quality control must ensure consistency in phage potency, genomic integrity, and absence of contaminants. These technical requirements significantly complicate phage manufacturing and increase production costs, especially when compared to conventional antibiotics [151].

In the European Union, phage-based therapies intended for veterinary use are currently regulated under the umbrella of novel therapies as defined in Regulation (EU) 2019/6. Such products must undergo centralized authorization by the European Medicines Agency (EMA), including rigorous assessments of safety, efficacy, and production under full Good Manufacturing Practice (GMP) standards. Although recent EMA guidance has introduced more flexible concepts such as “design space”—allowing for some variability in phage composition within approved bounds—these frameworks remain expensive and logistically demanding, particularly for large-scale aquaculture operations that require low-cost, high-volume treatments [145].

Some manufacturers have explored classifying phages as feed additives to bypass stringent medicinal product regulations. While this route offers a less burdensome regulatory pathway and does not mandate GMP compliance, it imposes strict limitations on phage formulation updates after approval. This restricts the ability to adapt phage preparations in response to evolving bacterial resistance, a key concern for the practical deployment of phages in dynamic aquaculture environments [145].

Magistral preparation—a strategy used in some countries such as Belgium—allows veterinarians to prescribe custom-formulated phage therapies in the absence of authorized alternatives. While this model may be suitable for individual treatments, it is not readily scalable to the demands of commercial fish farming, which typically involves administering therapeutics to thousands of animals simultaneously. Moreover, these preparations are bound by national frameworks and do not offer a viable route for broad market integration [145].

In conclusion, bacteriophages represent a promising tool for managing bacterial diseases in aquaculture, particularly as the sector seeks alternatives to antibiotics amid mounting concerns over AMR. However, to enable widespread adoption, it is essential to address production bottlenecks, develop scalable purification and quality control processes, and adapt regulatory frameworks to reflect the unique biology and use patterns of phages. Risk-based regulatory flexibility, along with supportive policies that incentivize innovation and surveillance against phage resistance, will be vital to unlock their full potential in aquatic health management [145,151].

### 4.3. Probiotics

Probiotics applied in aquaculture are derived from enzyme-producing bacteria and are administered through feed or to aquatic environments. They mainly improve fish immunity and resistance to diseases by synthesizing hydrolytic enzymes, improving the microbiota, and acting as growth promoters and immunostimulants [152]. Currently, a variety of probiotics have been used in aquaculture, including *Lactobacillus*, *Lactococcus*, *Enterococcus*, and *Bacillus* [152]. It has been found that *Bacillus rugosus* has strong auto-aggregation activity and coaggregation activity with *Aeromonas* and *Streptococcus*, therefore forming a barrier to prevent the invasion of pathogenic microorganisms [153]. In addition, *A. veronii* V03, as a novel pigmented probiotic strain, can improve the resistance of *C. carpio* to infection by *A. hydrophila* through enhancing fish innate immunity by boosting phagocytotic cells and antimicrobial enzymes [153]. Say et al. found that chitosan and other synbiotics derived from terrestrial crustaceans can enhance the efficacy of probiotics in the gastrointestinal environment of hybrid *Clarias sp.* catfish, with strongly increased serum immune parameters and immune chaperone gene expression [154]. In addition, by integrating the exogenous CRISPR-Cas system into the original probiotics, it is possible to achieve targeted cutting of multiple AMGs (*mcr-1*, *blaNDM-1* and *tetX*), thereby reducing the risk of AMG transfer [154]. However, AMGs and virulence factors have already been detected in commercial aquaculture *Bacillus subtilis* probiotics in China, which means that probiotics themselves may become potential carriers of AMGs, especially in high-density aquaculture environments where AMGs can be easily transferred [155]. The resistance of a potential probiotic strain to multiple common antibiotic categories is crucial in the selection process. Although the introduction of the CRISPR-Cas system has been proposed as a strategy to reduce the spread of AMGs, its biosafety and stability are still not fully verified.

### 4.4. Biosurfactants

Biosurfactants are amphiphilic mixtures of proteins, lipids, sugars, and phosphates produced by bacteria and yeast. They have antimicrobial, antiadhesion, and antibiofilm-forming activities [156]. A nontoxic glycolipid biosurfactant (BS-SLSZ2) derived from the marine *Staphylococcus lentus* exhibited antiadhesion activity and inhibited biofilm formation by preventing the initial attachment of cells to the surface. It also disrupted mature biofilms of *V. harveyi* and *P. aeruginosa*, thereby reducing receptor infection [156]. In addition, biosurfactant molecules can help fish grow faster through the secretion of enzymes and proteins, and display an inhibitory effect on some pathogens [157].

Lipopeptide-type biosurfactant extracted from a *Bacillus sp.* showed immunomodulatory activity in fish by increasing serum lysozyme activity and upregulating anti-inflammatory cytokines (IL-10 and TGF-β), enhancing resistance of *Labeo rohita* against *A. hydrophila* challenge [158]. Another characteristic of biosurfactants is that they have an affinity for both organic and aqueous phases and are highly biodegradable [159], making them useful for oil pollution control in aquatic environments. The biosurfactant prepared from peptides produced from fish liver and head waste displayed dispersion efficiency of up to 76.8% for Alaska North Slope oil [160]. Fish waste from aquaculture and slaughter therefore, has potential for further post-processing as a source of biologically and chemically active compounds.

Although biosurfactants are considered to have good biodegradability and environmental adaptability and can play a role in pollution control, their release into natural water bodies may disturb microbial communities and may even affect the balance of beneficial microorganisms in aquaculture ecosystems. Furthermore, the complex composition and diverse sources of biosurfactants have also brought a series of regulatory challenges to product standards, safety verification and environmental impact assessment [161].

### 4.5. Bacteriocin

Bacteriocins, which are widely present in various types of bacteria, are antibacterial peptides synthesized by bacterial ribosomes. They have antibacterial biological activity and are usually associated with the destruction of bacterial membrane integrity [162]. Advantages include degradability by proteases and being less harmful to the host or environment. Bacteriocin AS-48 from *Enterococcus faecalis* has an antimicrobial effect on the fish pathogen *Lactococcus garvieae*, eliminating it after 24 h at a ratio of *E. faecalis*: *L. garvieae* 1/10 CFU/mL [162]. *Paenibacillus ehimensis* NPUST1, a potential probiotic that was isolated from tilapia ponds, also has bacteriocin-like activity and shows antimicrobial efficacy against resistant *V. alginolyticus* and *V. parahaemolyticus* [163]. Administration of bacteriocin-producing probiotics has potential to be an effective solution to the problems caused by antibiotic use in aquaculture. However, the antimicrobial spectrum of most existing bacteriocins is narrow and not suitable as a widespread treatment of pathogenic bacterial infections in aquaculture. Bacteriocin-based combination (Pediocin PA-1 with citric acid and/or lactic acid) can enhance antimicrobial activity and broaden the spectrum of inhibition, as it was shown to reduce growth of *A. hydrophila* and *K. pneumoniae* synergistically [164]. The preparation process and biosynthesis expression system of bacteriocins vary greatly, resulting in significant differences in structure and stability of bacteriocins produced by different strains, which requires the establishment of an appropriate unified quality standard system and inspection methods [165].

### 4.6. Antimicrobial Peptides

Antimicrobial peptides (AMPs) are a class of small molecule peptides that are widely present in almost all organisms in nature. As an important component of the innate immune system, they have a broad range of antimicrobial activity against microorganisms such as bacteria, fungi and viruses, and are usually non-specific [166]. AMPs are mainly reported from fish epidermal mucus, acting as a physical or chemical barrier to resist invading pathogens [167]. Wang et al. found that compounds in flatfish skin mucus exhibited strong antioxidant, protease and lysozyme activity, which can show antibacterial ability against *E. piscicida* and *V. anguillarum* [168]. There are many types of fish AMPs, in which the most reported are α-helical amphipathic peptides. A new antimicrobial peptide (gcIFN-20) extracted from grass carp (*Ctenopharyngodon idella*) interferon 1 (IFN-Is) can form an α-helical structure and has strong bactericidal and anti-inflammatory activities [169].

**Figure 2:**
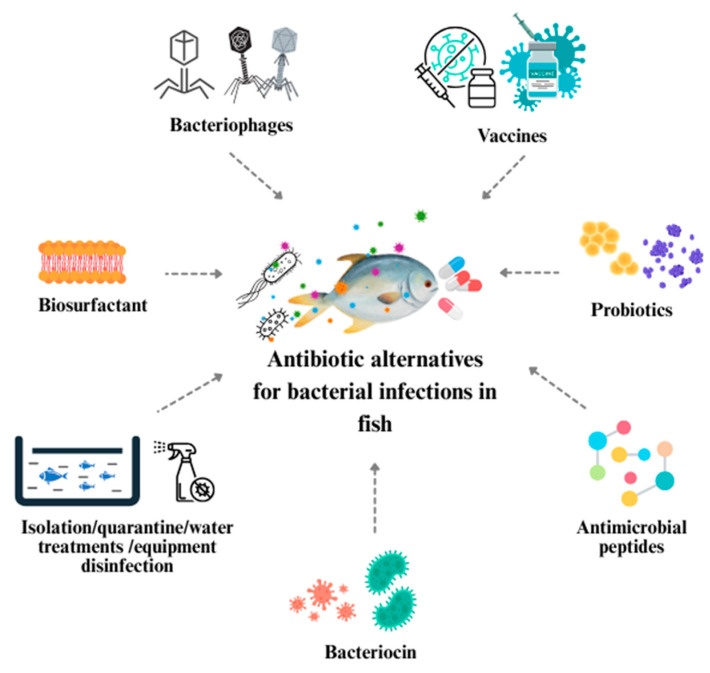
Antibiotic alternative therapies for treating fish bacterial infections (based on [125,126,127,128,129,130,131,132,133,134,135,136,137,138,139,140,141,142,143,144,145,146,147,148,149,150,151,152,153,154,155,156,157,158,159,160,161,162,163,164,165,166,167,168,169,170,171,172,173,174,175,176,177,178,179,180,181,182]).

Mutant AMPs constructed based on the naturally occurring 22nd amino acid AMP (Trematocine, Trem) in Antarctic fish *Chaitrematomus bernachaii* were found to target the ESKAPE group (*E. faecium*, *S. aureus*, *K. pneumoniae*, *A. baumannii*, *P. aeruginosa*, and *Enterobacteriaceae* family) [170]. In addition, hepcidin plays an important role in the immune response in fish as a cysteine-rich AMP. The derived peptide TroHepc2-22 synthesized from the mature peptide of hepcidin 2 from *Trachinotus ovatus* has good antibacterial activity against *V. harveyi*, *B. ichthyosporus*, *S. aureus*, and *S. agalactiae* by causing bacterial membrane rupture and cytoplasmic leakage, which also confirms its possible role in immunology [171]. Some AMPs not only have direct antimicrobial effects but can also modulate the host’s immune system. The fish-specific AMP piscidin-1 found in the gills and skin can play an immune-regulatory role when the external environment changes, and interact with the nervous system to enhance the fish’s resistance to disease [172]. Further research is still needed to optimize the dosage of AMP inducers in fish feed to prevent expression dysregulation. If the dosage is too high or the duration is too long, whether it will interfere with the fish’s own natural immune mechanism or have potential effects on the water microecology is still lacking systematic research [173]. The graphic (Figure 2) illustrates the range of antibiotic alternative therapies explored for treating bacterial infections in fish, based on references [125,126,127,128,129,130,131,132,133,134,135,136,137,138,139,140,141,142,143,144,145,146,147,148,149,150,151,152,153,154,155,156,157,158,159,160,161,162,163,164,165,166,167,168,169,170,171,172,173,174,175,176,177,178,179,180,181,182].

## 5. Current Considerations and Way Forward

Aquaculture plays a critical role in global food security, but disease outbreaks and the resulting reliance on antibiotics continue to fuel the spread of antimicrobial resistance (AMR). This review highlights several promising alternatives to antibiotics—including vaccines, bacteriophages, probiotics, biosurfactants, bacteriocins, and antimicrobial peptides—but their broader adoption remains limited by regulatory, technical, and economic barriers. Regulatory challenges are significant across all approaches. Autogenous vaccines lack harmonized quality control, while attenuated vaccines carry a risk of reversion to virulence. DNA and subunit vaccines, although safer, often require complex delivery systems and multiple doses. Phage therapy faces strict EU regulations under Regulation (EU) 2019/6, including EMA authorization and full GMP compliance—an expensive and time-intensive process. While alternative classifications (e.g., feed additives or magistral preparations) offer some flexibility, they also limit adaptability and large-scale application. Probiotics and bacteriocins face similar hurdles, especially concerning strain safety and consistency.

Economic and logistical factors further limit uptake. Advanced vaccines and phage preparations are often costly, require cold storage, or are not yet optimized for mass production. For small- and medium-scale farms, access to these solutions remains challenging. Field data on efficacy under real-world aquaculture conditions are often lacking, reducing farmer confidence and regulatory support. Monitoring and surveillance systems for AMR, phage resistance, and vaccine effectiveness remain underdeveloped in aquaculture, especially in the case of developing countries. Integration of aquatic disease data into broader One Health surveillance frameworks is essential to inform responsible policy and risk management.

To overcome these challenges, collaboration among researchers, regulators, industry, and policymakers is crucial. Harmonizing international regulatory guidelines, expanding public-private partnerships, and improving farmer education are key to enabling adoption. National and international funding bodies should prioritize the following:

- Scalable vaccine and phage delivery systems;
- Improved phage QC processes;
- Biosafety research for probiotics and bacteriocins;
- Adaptive regulatory frameworks tailored to aquaculture’s unique needs.

In summary, alternative strategies hold great promise for reducing AMR in aquaculture. However, translating innovation into practice requires coordinated action, regulatory reform, and investment in field-ready solutions. Only then can sustainable aquatic animal health management be achieved.

## Figures and Tables

**Table 1:** Literature-Based Analysis of Antimicrobial Resistance (AMR) and Antimicrobial Genes (AMGs) in Fish-Associated Bacteria.

Bacteria	Resistance Genes	Antimicrobial Susceptibility Test	Fish Species	References
*A. salmonicida*	*mcr-3*, *fox-2*, *cphA5*, and *OXA-427*	-	Brook trout and char	[52]
	*FOX-4*, *cphA5*, *blaOXA-427-like*, *APH[3″]-Ib*, *APH[6]-Id*, *floR*, *sul2*	Cefoxitin, chloramphenicol, florphenicol, tetracycline, oxytetracycline	Salmon	[54]
*A. hydrophila*	*act*, *aerA*, *alt*	Chloramphenicol, amikacin, gentamicin	Nile tilapia fish	[55]
	*sul1*, *strA-strB*, *aadA*, *bla*_TEM_, *bla*_SHV_, *tetA-tetE*, *tetM*	Ampicillin, streptomycin, kanamycin, nalidixic acid	African catfish and Pangasius catfish	[56]
*A. veronii*	*H-NS*, *bacA*, *mdtH*, *vatF*, *dfrA3*, *cphA3*	-	Mandarin fish	[58]
*V. harveyi*	-	Cephalosporin (vancomycin, cefoperazone, cefradine), aminoglycoside (piperacillin)	Spotted sea bass	[64]
*V. anguillarum*	*carB-19*, *mecB*, *novA*, *QnrS2*, *erm*, *sul4*, *catB9*, *optrA*, *cfr*	Penicillin, oxacillin, ampicillin, cefradine, neomycin, pipemidic acid, ofloxacin, norfloxacin	Rockfish (Sebastes schlegelii)	[65]
*V. anguillarum*	*qnrS*, *qnrB*, *StrAB*	Oxacillin, ticarcillin, streptomycin, ciprofloxacin	Korean mullets	[67]
*V. parahaemolyticus*	-	Ampicillin, amikacin, kanamycin	Yellowstripe scad, Indian mackerel, Black pomfret, catfish, Red tilapia	[66]
*V. vulnificus*	*QnrVC1*, *QnrVC7*, *tet*R, *tet*B, *bla*CTX-M-55, *tet(59)*, *sul2*, *QnrVC5*, *gyr*A, *gyr*B, *par*C	Amoxicillin, Colistin sulfate, Metronidazole, Streptomycin, Clindamycin	Asian sea bass	[16]
*V. cholerae*	*-*	Streptomycin, ampicillin, rifampicin	Bighead carp, Goldfish, Grass carp, Channel catfish, Longface emperor, Northern snakehead, White Amur bream, Turbot	[69]
*S. iniae*	*ermB*, *tetM*, *tetO*	Erythromycin, tetracycline	Nile Tilapia	[72]
*S. agalactiae*	*ermB*, *tetM*, *gyrA*, *par*C	Erythromycin, tetracycline, enrofloxacin, penicillin	*Schizothorax prenant*, *Schizopygopsis pylzovi*	[74]
*S. parauberis*	serotype Ia: *erm(B)*, *tet(S)*, *ANT (6)-Ia* serotype II: *tet(M)*, *mef(J)-msr(I)*	Amoxicillin, Oxytetracycline, Erythromycin	Olive flounder	[76]
*P. putida*	*-*	Piperacillin-tazobactam, Cefepime, Amikacin, Levofloxacin, Amoxycillin	Rainbow Trout	[83]
*P. aeruginosa*	*tetA*, *tetD*, *tetM*, *sul1*, *bla*_CTX-M_, *bla*_TEM_, *bla*_SHV_	Oxytetracycline, co-trimoxazole, doxycycline, enrofloxacin, ciprofloxacin, cefotaxime, ceftazidime, and ampicillin	Rohu, Catla, Pangasius	[84]
*P. fluorescens*	*-*	Piperacillin, ceftazidime and cefepime	Nile Tilapia	[86]
*P. baetica*	*mexF*, *aac(6′)-31*, *qacH*, *bla*_OXA-2_, *qacEΔ1*, *sul1*, *tet(A)*	Ceftriaxone, cefotaxime, cefoxitin, aztreonam, florphenicol, tetracycline, oxytetracycline	Salmon	[54]
*F. columnare*	*bla*_TEM_, *bla*_SHV_, *tetA*	Pencillin, cephalosporin, aminoglycoside, nitrofurans, polymyxin B and tetracycline	Nile Tilapia	[120]
*F. psychrophilum*	*AGly*, *bla*, *col*, *flq*, *gly*, *mls*, *oxzln*, phe, *sul*, *tet*, *tmt*	-	Ayu, Stickleback, Rainbow trout, Coho salmon, Atlantic salmon	[90]
*A. baumanni*	*bla*_CITM_, *bla*_SHV_, *tetA*, *qnrA*, *bla*_VIM_, *aac(3)-IV*, *sul1*, *dfrA1*, *qnr*	Tetracycline, ampicillin, gentamicin, erythromycin	Fish market	[100]
*A. pittii*	*-*	Cephalexin, cefoxitin, nitrofurantoin, ampicillin, oxacillin, penicillin-G, bacitracin, and trimethoprim	Rohu	[101]
*A. lwoffii*	*-*	Florfenicol, sulfadiazinum, penicillin, tetracycline	Hybrid sturgeons (*Acipenser baerii*♀ × *Acipenser schrenckii*♂)	[121]
*A. johnsonii*	*qacED1*, *qnrS*, *sul1*, *dfrA*, *aadA1*	Ampicillin, gentamicin, lincomycin, nalidixic acid, tetracycline, oxytetracycline	Nile tilapia	[122]
*E. ictaluri*	*floR*, *tetD*, *sul2*	-	Channel catfish	[108]
*E. piscicida*	*floR*, *tetA*, *tetR*, *sul2*, *aph(6)-Id (strB)*, *aph(3)-Ib (strA)*	Florfenicol, chloramphenicol, oxytetracycline, doxycycline, erythromycin, tetracycline, azitromycin, spectinomycin, sulfonamide, and bacitracin	Hybrid catfish (channel catfish × blue catfish)	[111]
*E. tarda*	*bla*_TEM_, *sul1*, *tetA*, *bla*_CTX-M_, *aad1*, *qnrS*, *qnrA*	Ampicillin, amoxicillin, tetracycline, trimethoprim-sulphamethazole, cefotaxime, streptomycin, gentamycin, ciprofloxacinand, enrofloxacin	Nile tilapia and African catfish	[117]
Methicillin-resistant Staphylococcus aureus (MRSA)	*femA*, *blaZ*, *tetA*, *tetM*, *ermB*	Erythromycin, ampicillin, rifampicin, clindamycin	Marine aquaculture fish	[118]
Extended-spectrum β-Lactamase (ESBL)-producing Enterobacterales	*bla*_CTX-M-15_, *bla*_CTX-M-27_, *bla*_CTX-M-55_	Ampicillin, tetracycline, trimthoprim-sulfamethoxazole, chloramphenicol, nalidixic acid, streptomycin, and ciprofloxacin	Edible river fish	[119]

## Data Availability

Data sharing is not applicable to this article as no datasets were generated or analyzed during the current study.

## Abbreviations

The following abbreviations are used in this manuscript:

AMU	Antimicrobial use
AMR	Antimicrobial resistance
AMG	Antimicrobial resistance gene
ARB	Antibiotic-resistant bacteria
MDR	Multi-drug resistance
MRSA	Methicillin-resistant *Staphylococcus aureus*
ESBL	Extended-spectrum β-lactamase
